# Multicultural desires? Parental negotiation of multiculture and difference in choosing secondary schools for their children

**DOI:** 10.1111/1467-954X.12076

**Published:** 2013-09-18

**Authors:** Bridget Byrne, Carla de Tona

**Affiliations:** 1 University of Manchester

**Keywords:** class, ethnicity, multiculture, multiculturalism, place, race, school choice

## Abstract

This paper considers the ways in which parents talk about choosing secondary schools in three areas of Greater Manchester. It argues that this can be a moment when parents are considering their own attitudes to, and shaping their children's experiences of, multiculture. Multiculture is taken as the everyday experience of living with difference. The paper argues that multiculture needs to be understood as shaped not only by racialized, ethnic or religious difference (as it is commonly understood) but also by other differences which parents may consider important, particularly class and approaches to parenting. We stress the need to examine what parents say about schooling in the context in which they are talking, which is shaped by local areas and the experiences of their children in primary schools. Based on interviews with an ethnically mixed groups of parents from different schools, we show how perceptions of the racialized and class demographics of schools can influence parents' choice of secondary schools. The paper also argues that attention needs to be paid to the ways in which terms such as ‘multicultural’ and ‘mix’ are applied uniformly to very different contexts, be they particular schools or local areas, suggesting there is a paucity of language in Britain when talking about multiculture.

## Introduction

There has been an increasing trend for those from both the left and right to pronounce on the death/decline/end of or need to ‘kill off’ multiculturalism. Trevor Philips (the chair of the then Commission for Racial Equality in the UK) controversially declared the failure of ‘multiculturalist’ policies in 2004^1^ (an issue which has also repeatedly been discussed by *Prospect* magazine^2^ among others). These policy discussions need to be understood in the context of a post 9/11 ‘war on terror’ and also as shaped by responses to the disturbances in Britain in 2001 which focused on a perceived rise in segregation (particularly in cities of northern England with large Muslim populations) (Cantle, 2001; Phillips *et al*., 2008). Thus, the debates around multiculturalism have tended to have an increased attention to religious difference (particularly Islam), alongside the older focus on racial/ethnic difference. In February 2011, David Cameron, the Prime Minister launched a ‘war on state multiculturalism’^3^ which he blamed for encouraging separatism and therefore being responsible for the radicalization that can lead to terrorism. One ‘front’ of this war for the Prime Minister was to ensure that schools teach a ‘common culture’. It is not unusual that schools are a focus for critiques/promotion of policy around multiculturalism. However, despite the perhaps premature declarations of its demise, there exists very limited agreement on what multiculturalism actually is. Stuart Hall has argued that we need to distinguish between multiculturalism and multi-culture. For Hall, the multi-cultural is a description of the social characteristics which arise when different cultural communities live together, whilst multiculturalism is a form of governance, involving strategies and policies which attempt to manage diversity and multiplicity in multicultural societies (Hall, 2000: 209). As Ralph Grillo (2007; see also Pitcher, 2009) argues, in debates over multiculturalism, what is often being opposed is a strong form of multiculturalist policy which only rarely exists, rather than the weaker form of small accommodations which has actually been implemented in Britain.

Wise and Velayutham argue that the sociological literature often fails to ‘deal adequately with the everyday lived reality of cultural difference in superdiverse cities and spaces’ (2009: 2). This is what Paul Gilroy calls the ‘unkempt, unruly and unplanned multiculture’ which has emerged in British civil society (2004: 2). This article provides a sociological examination of one site of negotiation with multiculture in order to explore how it is lived in the everyday rather than reproduced in political and policy rhetoric. The focus in this paper is on schools as sites of mix and multiculture. We are not concerned here with the experiences of, or interactions between, children or teachers in the school playground or classroom. Rather our interest is in school choice as a site of parental negotiation with different ideas of multiculture. How does multiculture figure when parents are choosing secondary schools that will be ‘right’ for their children? This article takes school choice as one point where racial, cultural or religious difference may (or may not) emerge as a difference that matters. Drawing on interviews with parents in selected areas of Greater Manchester and conducted at the time when they were applying for secondary school places for their children, this paper asks how they approach the social worlds which a new school represents for their children. How do they think about and try to shape their children's encounters with multiculture? Talking to parents about school choice is a particularly interesting site to explore approaches to multiculture, not least because of its emotional content for parents. As one parent who we interviewed put it: ‘it feels like you lost contact with your children, they're left to the whole wide world’. Parents approach schools as a place of sociality which will both shape their children and in which they want their child to be comfortable. It is here that questions of cultural mix and multiculture can become critical, but also may be accompanied by, or over-ridden by other concerns, such as those around class.^4^

The research is distinctive from much other work on multiculture/multiculturalism in three ways. First, we are drawing on the experience of a range of parents with different racialized identities, rather than, as is often the case, focusing on only the white majority response to a multicultural ‘challenge’ which is often framed as if coming from ‘outside’. In addition, we are alert to the ways in which everyday multiculture is experienced as classed as well as raced. That is, we have found that when parents are thinking about school choices in terms of the differences that their children might encounter, questions of class are raised perhaps as much as race/ethnicity and in ways in which it may not be productive to separate the analysis. Also, it is important to understand how responses to cultural differences are focused on religious difference as much as being explicitly expressed as concerned with ethnicity or race. Finally, the article highlights the importance of *placing* a study. The everyday is lived in particular places in different ways and the experience of multiculture is shaped by the places in which it occurs as well as the routes by which people come to find themselves in those places, routes of birth, of choice, happenstance, or placement by the state.

The article will first discuss the sociological literature on multiculturalism and on school choice. Having introduced the study, we will explore the different range of talk produced by interviewees when considering questions of place, schools and difference. The article will argue that a range of discursive repertoires for talking about difference, both in terms of local schools and local areas are discernible. There are a variety of responses to what differences matter, and these responses show that it is not just racialized difference that matters. Religion may feature as an important marker of difference, albeit closely associated with racialized differences. Equally, class may arise as the most important consideration when thinking about who parents want their children to socialize with.

## Multicultural policy debates: education and school choice

Anne Phillips (2007: 3) argues that in the early 21st century: ‘Multiculturalism became the scapegoat for an extraordinary array of political and social evils, a supposedly misguided approach to cultural diversity that encouraged men to beat their wives, parents to abuse their children and communities to erupt in racial violence’, and she could have added the threat of terrorism. If the current policy climate persists, particularly with the close ties being drawn between debates on multiculturalism and concerns about both immigration and terrorism, it would seem that we are experiencing a distinct ‘turn’ in terms of government policy towards religious, ethnic and racial difference. This turn draws to a close the era initiated by Roy Jenkins in the 1960s. Roy Jenkins, as the Labour Government's Home Secretary, argued for a move away from an assimilationist approach towards an understanding of ‘equal opportunity accompanied by cultural diversity in an atmosphere of mutual tolerance’ (see Grillo, 2007). In the context of education, this approach was developed in the Swann Report of 1985 *Education for All* which argued for cultural pluralism and opened the door for a range of muliticultural policies from schools and LEAs (see Rattansi, 1992). Schools have played an important role in the politics of multiculturalism where they have generally been seen as a site both of tension and risk around culture and diversity but also as a possible solution to racialized prejudice and inequalities. As Rattansi argued, ‘the basic educational prescription [of multiculturalism] is the sympathetic teaching of “other cultures” in order to dispel the ignorance which is seen to be at the root of prejudice and intolerance’ (Rattansi, 1992: 24). However, many anti-racists criticized the multiculturalist approach for failing to address racist structures and ideologies and assuming that a ‘dose’ of ‘other cultures’ – often read as religion (Yuval-Davis, 1992: 283) – would cure ignorance and thereby end racism (Hesse, 2000).

This is not to say that ‘multiculturalism’ consists of a coherent political or policy discourse (as Stuart Hall, 2000, argues, it should properly be understood in the plural). Lentin describes it rather as ‘a patchwork of initiatives, rhetoric and aspirations’ (Lentin and Titley, 2011: 2). Nonetheless, despite its relatively modest ambitions and partial implementation, it seems clear that we are facing what Derek McGhee describes as the ‘systematic dismantling of multiculturalism as the organising rhetoric of public policies’ (McGhee, 2005: 1.4). The discourse within education has shifted from multiculturalism to ‘active citizenship’ (Yuval-Davis, 2007). In this shift, schools are again central to the attempt to create a new form of citizenship based around the elusive ‘common culture’ of the nation in a reframing of cultural debates within what Clare Alexander argues is a ‘reinvigorated nationalist discourse’ (Alexander, 2007: ii).

Running alongside the attention given to multiculturalism in schools has been an increasing marketization of the educational field, with parents constructed as choosers or consumers in the field (Gabriel, 1994; Ball, 2003). Studies have tended to focus on classed (particularly middle-class) practices of educational choosing and the desire for ‘people like us’ without sufficient attention to how these practices are racialized (see Ball, 2003; Croft, 2004; Devine, 2004; Bruegel, 2006; Burgess *et al*., 2006; Raveaud and van Zanten, 2007). While white middle-class parents are overwhelmingly represented in this literature, their whiteness is often unmarked and race talk and practice are left unexamined (Byrne, 2009). An important exception to this tendency is Diane Reay and her colleagues who have explicitly examined the experiences of white middle-class parents in choosing ‘mixed’ inner city schools. For some she argues that these choices serve to display their liberal credentials and secure their class position: mixed schools can offer a form of ‘multicultural capital’ (Reay, 2008: 91; Reay *et al*., 2011).

Some studies have highlighted how ethnic minority middle classes share many of the parenting practices and strategies of the white middle-class counterparts (see Lareau and McNamara Horvat, 1999; Archer, 2010; Ball *et al*., 2002). However, there are complex racialized elements that set their experiences apart from those of the white majority. For example, there is evidence that schools are more accepting of white parents' assertiveness than minority ethnic parents, who also feel more ambivalent and reflexive and ultimately less confident in adopting the ‘complaining parent persona’ (Archer, 2010: 465). Debbie Weekes-Bernard (2007) argues that ‘the current education market and its promotion of increased parental choice may require parents from some BME communities to engage in processes of “flight”, which may result in their children being educated in schools, and their families living in residential areas, where they form a clear minority’. The same parents were often worried about their children suffering increased racism as a result (see also Gillborn *et al*., 2012 and Archer, 2011).

The literature on school choice has also largely focused on London and the south-east of the England. By contrast, in this paper, we document the experiences of those living in Greater Manchester, one of the most diverse cities in the north-west of England. We reflect on these issues looking at the experiences of diverse groups of parents whose children were in Year 6 of primary school, and who were in the process of choosing secondary schools^5^ in the Greater Manchester area. Our analysis focuses on both white and ethnic minority parents. We examine parents' motivations about their choice (or lack of choice) of schools, setting out to explore what their narratives tell us about contemporary forms of both classed and racialized mixing and separation and the talk which surrounds it. This is also placed within the context of local schools and local areas. As Cross and Keith (1993: 9) argue: ‘race is a privileged metaphor through which the confused text of the city is rendered comprehensible’.

## The study

The research on which we draw is a 12-month study funded by the Economic and Social Research Council, on secondary school choice and the intersection of race, ethnicity and class in influencing parental choice, in three areas in Greater Manchester: Cheadle, Chorlton and Whalley Range. These areas were selected because of their socially mixed populations and different class and ethnic compositions. Cheadle is the most affluent and the least ethnically diverse area, Chorlton is ethnically mixed with a large middle-class population (particularly public sector employees), and Whalley Range more ethnically diverse and with a higher number of working-class residents. Ward level statistics from the 2001 census show that 87 per cent of Cheadle residents, 79 per cent of Chorlton residents and 48 per cent of Whalley Range residents were white. Twenty-two per cent of Whalley Range residents were Pakistani Asian and 8 per cent black British; 24 per cent of the population of Whalley Range were born outside the United Kingdom, compared to 9 per cent of those from Chorlton and 7 per cent from Cheadle (Office for National Statistics, http://neighbourhood.statistics.gov.uk). These demographic characteristics were broadly reflected in the school populations. So, in terms of the primary schools^6^ which the children were already attending, in Whalley Range (Heath Primary School), the large majority of children came from ethnic minorities and there was a much higher than average take up of free school meals.^7^ In Chorlton (Longford Primary School), just over 50 per cent of the children were from ethnic minorities and there was a below average eligibility for free school meals. Finally, in Ashover Primary School in Cheadle, under 25 per cent of the children were from ethnic minority background, few of whom were at an early stage of English and there was a lower than average eligibility for free school meals.^8^

It is clear that the schools cannot be simply read off as representing the larger areas in which they are found.^9^ So, for example, taking just one characteristic, Whalley Range has a population where 48 per cent are white, whilst at Heath Primary school only 11 per cent of students are classified as white. Equally, whereas the ward level statistics show 79 per cent of Chorlton residents are white, at Longford Primary, this figure is 51 per cent. For Cheadle, the figures are 87 per cent of residents are white whilst 76 per cent of students at Ashover Primary School are white. The differences between area statistics and school-level ones will be influenced by different age demographics for the different ethnic groups. But they will also be influenced by parental choice of primary schools and it may be that there is an element of ‘flight’ in Whalley Range, by parents who sent their children to schools in neighbouring areas.^10^ The school also draws pupils from neighbouring areas with high numbers of ethnic minority areas (such as Moss Side). Thus whilst the schools do not represent the areas in a straightforward way, we are interested in considering how parents' responses to questions around school choice can also be understood as part of their experience of their children already having attended particular schools and living in particular areas. Whilst the schools may not have exactly matched the demographic make-up of different areas, experiences of going to particular schools also played a role in shaping interviewees' perceptions of the area in which they lived.

Between October 2009 and March 2010, we conducted 47 semi-structured interviews with 54 participants,^11^ who were parents of Year 6 and in one case of Year 5 children and whom we contacted while participating in parents' evenings at the primary schools. Of the 54 interviewees, 29 were white parents (of whom one was Jewish and one woman had mixed-race children) and 25 were from racialized minorities (15 were Asian parents, 4 Afro-Caribbean parents, 5 were of Chinese, Iraqi, Somali, Libyan origins respectively and 1 was Turkish). In Cheadle, we interviewed 15 white and 4 ethnic minority parents; in Chorlton, 11 white and 9 ethnic minority parents; in Whalley Range, we interviewed 4 white and 11 ethnic minority parents. Our research participants ranged in age between 25 and 55 years, had different levels of formal education and professional qualification. To a certain extent, it was a self-selecting group as these parents were attending teachers' meetings and had demonstrated their involvement in their children's schooling. The vast majority of the interviewees were women, with only 10 men taking part in the research, and of those most were interviewed as part of a couple. This is perhaps partly a feature of women being more amenable/available to be interviewed^12^ and also may reflect a greater involvement in schooling by mothers rather than fathers (David, 1997; Ball, 2003).

The interviews ranged from about 40–100 minutes and were conducted mostly in the interviewees' homes by either Carla de Tona or Bridget Byrne. The interviewees were asked when and why they had moved into the area that they lived in, and about their experiences of choosing primary schools for their children. They were then asked about what they had done to find out about secondary schools, how they had made their selections and who had been involved in the decision (children, partners etc.). They were also asked about what they thought of the process of choosing schools and how they thought their children's schooling compared to their own. At the end of the interview, if questions of the social and ethnic make-up of the schools they had chosen had not already arisen, then they were asked about this. For this article, a thematic analysis was undertaken, looking particularly at how and when in the interview issues about the demographic make-up of the school and the local areas were raised by the parents. This was done across the whole body of the transcriptions, but also with attention to how different groups (either defined by the areas in which they lived or by similarities such as ethnic origin) might produce talk which was drawn from common discursive repetoires.

It was clear from this analysis that it was far more likely for questions of race to emerge unprompted in the interviews with ethnic minority parents, whereas white parents were more likely to discuss the issue only when they were directly asked about it. The main exception would be some of the white parents of children in the school in Chorlton where, as we shall see, there was a strong discourse of positive perceptions of multiculturalism in discussing the area and the school. This study sheds light on the ways in which parents (drawn from different areas and with experience of different primary schools) talked about questions of difference and diversity and their reflections on their desires and fears for their children and their schooling. This contributes to sociological understandings of the ways in which multiculture is enacted as well as suggesting that study of multiculture needs to be attentive to the workings of discourses of class and religion as well as race and ethnicity. It is clear that whatever the fate of multiculturalism as public policy, in everyday lives, a complex range of categories of cultural difference and sameness are employed.

## Embracing multiculture

In terms of what could be characterized an orientation towards multiculture, it would seem from the interviews that there are broad, area/school-specific discourses circulating. Thus, parents of children in the school in Chorlton and to a certain extent Whalley Range have a particular range of responses to ideas of cultural difference which differ from those of the Cheadle school where the talk tends to be more fearful and tentative in its embrace of mix. This might be expected from the fact that the parents of children in Chorlton and Whalley Range schools all appeared happy with their children's current primary schools which had high levels of ethnic, religious and to a certain extent, class diversity. Many of them stressed the ‘comfortable’, ‘friendly’ or ‘nice’ atmosphere they found in the schools their children attended. This orientation towards multiculture or everyday conviviality (Gilroy, 2004) is shared by white and non-white respondents and of people in different class positions in these areas to a large degree, but with some significant differences. In the case of the parents from the Chorlton school in particular, it is also accompanied by a sense of a liberal and sometimes ‘alternative’ lifestyle which has classed characteristics, also shared by white and ethnic minority respondents.

Sara, a white social worker, whose daughter goes to school in Chorlton tries to explain that there is a kind of ‘Chorlton talk’ around diversity which she suggests enforces certain silences. The sensitivity of the issue is perhaps expressed in her false start – she tries to first say what might *not* be desired (otherness) but fails and switches to an alternative, positive phrasing of the issue:

you wouldn't have somebody in Chorlton saying, “I don't” … “I want my children to go to a school where people have nice families and that they are white, and nice middle [class]” … […] that might be what they felt, but you wouldn't openly say it.

Certainly, what we heard most frequently in the interviews with respondents in the Chorlton school was a positive response to diversity, but one which also had a broad approach to the notion of difference, often encompassing questions of ‘ethical approach’ (to child-rearing or, for example, vegetarianism) as well as questions of sexuality and cultural difference. This highlights an important gap in the literature around multiculture which often fails to address how racialized or religious differences are understood as embedded within a range of different ‘lifestyles’ or ‘cultures’, which are also classed. Which differences matter at a particular moment is therefore also likely to shift according to a range of considerations and the geographic location which is being considered.

Fran (a white health worker) explained how she chose the primary school for her child:

well, I think because we're not religious, we didn't want any sort of Christian school or anything like that. We wanted a school that had a good mix of different kinds of kids, so Longford School is quite a mix of like different cultures. There's Asian kids, Afro-Caribbean kids, Chinese kids, there's loads of different kids and there's quite a lot of gay parents as well, so we wanted the kids to get a really good idea of difference basically.

This embrace of diversity was shared by Jas and Tej (British Asian managerial employees) who together explained how they chose Longford Primary school:

Jas: ‘Tej spoke with other parents and they thought Longford was a good cosmopolitan school’

[…]

Tej: ‘we found it was more of a community school. You know… . the mix, the parents were quite similar to what we wanted to be involved with, you know. It is quite relaxed, like there is no uniform. Children are from all different backgrounds … there is a good network, the parents' ethics are quite good.’

Jas and Tej were comparing this school with the one (in Whalley Range) that their son initially went to which they characterized as ‘90 per cent Asian’,^13^ so the mix is critical. Jas and Tej were both Sikhs with ancestry from India and the school they were referring to would have had children from predominantly Pakistani Muslim backgrounds, so religion as much as ethnic origin is likely to make up a significant part of the mix that matters in this situation. This question of getting the mix right was shared by many of the respondents and is similar to the findings of a study of discourses produced by white parents in some areas of South London (Byrne, 2006).

Wrapped up in the expressed desire for ‘mix’ may be different concerns and experiences. For many, the desire is presented pragmatically as gaining ‘real life’ experience for their children. This could be regarded as the same kind of desire for social and cultural capital that Diane Reay *et al*. (2007) found in their study on white middle-class parents. However, it is interesting to note that it is a desire shared also by non-white parents. As Serena, a nurse whose parents migrated to Manchester from the Caribbean, and whose children went to the Chorlton primary school argued:

Children nowadays need to know everybody, before they make up their mind about a certain class of people or a certain ethnicity of people, they need to know everybody, and I think that our children now that are growing up are more, are much more, are much better in communicating with different types of people, than say [we] were or you know people were a couple of years ago or before.

This desire is expressed in very similar terms by Kelly, a white teacher whose child went to Longford Primary in Chorlton: ‘We want our children to grow up knowing there's lots of different kinds of people around and they're all … it's all good.’

## Safety in numbers

However, within a similarly expressed desire for mix, the different experiences of individuals within a racialized context did produce different concerns (which mirror those found for applicants to higher education see Ball *et al*., 2002). So for some, experience of racism was presented as causing their desire for ‘mix’, as Nasreen who had come to Britain from Bangladesh as a child and worked as a translator, explained: ‘obviously diversity means a lot because I was – I went to a school where it was all white. And there was a lot of racial abuse so – which is something that I do not wish for my daughter. So if there is a school where it's quite diverse hopefully she won't get that.’ This explanation is combined with the more general desire for mix: ‘all you wish for your child [is] to grow up aware of other cultures and religions. And not just cultures and religions, different experiences of lifestyle.’ A similar worry about racism and the desire for ‘safety’ in diversity was expressed by Sabah a British Pakistani nursery worker:

even though I'm not racist or anything that you think about your child if he's going to be the only Asian in the whole school, how is he going to, how are other children going to react to him? And then you think about that, I sometimes, … . It might be fine you just, it's your worries at the back of your mind I think. I think yeah you do opt for the safer choice. I think as parents we do go for the safer choice. So where we see that … you know the way it is diverse and it's got a lot of mixture of children.^14^

It is clear that, for these parents, talking about ‘mix’ includes consideration of religious difference as well as racialized differences.

For some parents from ethnic minority backgrounds, there was also a sense that other (white/Christian) children would benefit from ‘mixing’ with their children. Halima sent her first child to an Islamic school but her other children have gone to Longford Primary School in Chorlton: ‘I must admit I've changed my views since then’. She likes the way all faiths are introduced to the children:

I am glad that they've been able to interact with other non Muslims. Because it's good, because we see so much stereotype of Muslims on the media, and I don't want that stereotype to have that [effect]. […] I don't want my children to be like cocooned in a school where they wouldn't be able to interact with other children.

Some white parents also qualified their approach to a ‘mixed’ school by suggesting there was a point at which their child became too much of a minority, which might be characteriz ed by class, race or religion or a complicated combination of them all:

We'd heard that it [a potential alternative school] was predominantly an Asian school so thought it might be quite difficult for a white child to fit into a really predominantly Asian kind of culture. And Longford has like so many different cultures, I just thought it'd be better for the kids if they had a really good mix of children and not just one predominant like White, Asian, whatever in that school. (Fran, white, nurse).

I supposed that might have influenced me in thinking are there, is she going to have other children who, you know that she's going to have similar things in common with, lifestyles and backgrounds as well as other people. So more of a mix, so it's not about, erm, I just want, I would like her to go where there's a mix of children. … I don't want her to go to school where it's all white children, but I wouldn't want her to go to a school where it's all predominantly Asian children either, just because I think, you know it's nice for there to be a mix. (Terri, White health visitor).

Also underlying much of the talk of the parents from the Chorlton school was an equivocal relationship between desire for ‘mixture’ in terms of race and questions of class (Skeggs, 2004). Many of the parents share a hesitation around mixing with a classed other. In part this suggests the power of racialized discourses around the ‘white working class’ (Sveinsson, 2009). It did appear that class was an important concern, particularly when it came to thinking about secondary schooling. So Ken, a white courier whose children went to Longford School, expressed a positive desire for a multicultural mix, but he also said he was concerned to keep his daughter ‘away from the rabble’:

Well it's very much the two tier thing that we said – there's this end of Chorlton where it's all middle class and there's the other end of Chorlton near the school where there's a huge … council estate and it's definitely a two tier feel to it.

Kelly, a white primary school teacher living in Chorlton demonstrates again the interplay between race and class in talk around diversity where cultural difference, when it applies to a racialized or ethnic difference is celebrated. However, responses are more equivocal around class:

There's a really good mix at the school. Lots of different backgrounds, lots of different races, lots of different languages spoken there, lots of different you know, sort of home lives they've come from, different kinds of families and it's sort of well celebrated there. The children grow up very tolerant of people's differences. But I suppose a lot of people in Chorlton are sort of teachers or social workers or you know those sorts of professions and well … I think when you have your kids first of all you can be a bit snobby about wanting them to have friends whose parents you like which tells, it doesn't sound very nice now (laughs) but you … I think you want your children to have friends to choose from that are going to be nice people to play with.

Educational policy on diversity tends not to refer to class differences or ‘working classness’ as something that might be celebrated and may also impact on how parents view such differences. It would appear that, for Kelly, the markers of who is not ‘nice’ (and not to be ‘celebrated’) would be shaped more by class than ethnicity or religion. However, for some of the parents, class and race were equally part of the mix that was desired. Rebecca, a white local government officer explained what she liked about the local high school:

There's things that I like about Parkside High which is that it's really mixed, it has children from lots of very different kinds of backgrounds and it seems to bring them together in a really positive way […] although it wasn't the reason I chose that school, it's something that's really strong. … there's something about kids that come from there that feel like that confident in being themselves and not just going with the crowd.

Rebecca then went on to explain the impact she felt going to Parkside High had had on her eldest daughter who was now at university and who maintained good friendships with her school friends:

And they are a really mixed group. Some of them are at university, some are working and done different things with their lives, but they're still really good friends, a mixed group class wise and ethnicity wise. But the people that she sees at university are a very narrow group of people who've mostly come from private schools, mostly come from very narrow social backgrounds.

None of the parents interviewed in any of the areas had applied (or intended to apply) to private schools for their children (some had applied to grammar schools and the one academy which was in the Whalley Range and Chorlton area). For some there was a concern that their child would be unable to keep up with the lifestyle of other children whose parents were more prosperous. There were also objections to the idea of selection on the basis of wealth. As Rebecca a white government officer living in Chorlton put it:

I think, well, we live in a community full of lots of different people, so why on earth would you want to separate out groups for your children's education? Part of growing up is mixing with people from wherever.

## Questioning multiculture

An interesting issue which arises out of this research is that we lack a highly developed vocabulary for describing diversity. Thus, in all three areas, which are quite distinct from each other in terms of both the residential population and the make-up of the schools, very similar phrases are used. So Cheadle, Chorlton and Whalley Range are all described as areas with ‘a good mix’, ‘very diverse’ and ‘very multicultural’ by different respondents. But what diverse or mixed means will vary in each case. It is often difficult to read off from people's description of the ‘mix’ what that actually means in demographic terms. However, whilst the parents from the Cheadle school, which was notably whiter in terms of residence and school intake than Chorlton or Whalley Range, did express a desire for ‘mix’, there was also a more explicit expression of fear and a risk of something lost. This outlines perhaps what might be seen by these parents as the limits to the ‘tolerance’ of difference. Much of the fear expressed is framed around religious as much as ethnic difference, although it is clear that the two are mutually constituted. It is possible to discern a different discursive field in operation among the parents of the school in Cheadle. Thus, where Sara above suggested that there are things that are almost unsayable in Chorlton, they were expressed more freely by parents in the Cheadle school. What appears to be under threat is a sense of Christian culture. However, it would be mistaken to see this as a straightforward replacement of ethnicity or race by religion. Often, the desire for Christianity (or conversely the fear of or concern about the Muslim) is coupled with ethnic or racialized descriptors. For instance, Natalie a white office worker, explains why she likes Ashover Primary School in Cheadle (she is one of the few white interviewees from Cheadle to bring up questions of racialized difference without prompting):

because of its ethics. … It was quite a Christian background actually, although there were children from other denominations that came to the school, it was a predominantly white Christian background that the children came from which I actually quite liked. I'm not an overly religious person.

Although a desire for a ‘predominantly white Christian background’ appears to be sayable in the Cheadle context in a way that it might not be in Chorlton, there remain other restrictions on what is acceptable to say, at least without care. So, as Natalie goes on to explain how the demographic nature of Cheadle has changed since the building of a mosque in the area about which she and her husband ‘weren't overly happy’, she says ‘I have to be careful what I say here’. Natalie also explains some of the limits to easy mixing which she has experienced: ‘And you – to a certain extent you're still segregated ‘cos we don't know and you do tend to put up barriers. You do become defensive with what you don't know.’ This segregation also translates into concerns about schooling:

The only thing that concerns me over ethnic backgrounds that go into secondary school is – and you do get it – is to a certain extent gang culture because again, because there is still a barrier and there's always going to be a barrier […] a lot of the time you'll get the Asian children sticking together and the white children sticking together. It causes tension.^15^

Natalie expresses some discomfort in talking about ethnic and religious difference and worries about her own ignorance. Similarly, Sharon a white civil servant also from Cheadle, referred to British Asian families as ‘white Asian’. She said:

I just know an Asian person from the colour of their, you know from [what] they look like … it's, but I don't know whether they are *white* Asian or whether they're not.

Sharon appears here to be confusing Asian for Muslim (or alternatively a British Asian has been rendered white) which again suggests that ‘diversity’ talk is less current. It also points to the intertwining of religion and ethnicity in her perspective. Sharon also draws on familiar discourse which is anti-multiculturalism or an understanding of ‘political correctness’:

There's been a lot of ill-feeling in the past few years … with parents that celebrate a lot of the Asian holidays and stuff but we're not allowed to celebrate any of ours because it's classed as racist. We're not allowed to celebrate St George's Day and … they stopped two children sending Christmas cards out and there's been a lot of stuff like that. […] There are a lot of subjects that white British children are not allowed to learn about because the other pupils' parents say it goes against their religion for them to learn about it and so they suffer in that respect.

This narrative was shared by Melanie, a white beauty therapist also from Cheadle. Again, however, Melanie feels she has to choose her words carefully, we don't know what word she wants to say, but it clearly is difficult:

Interviewer: How would you characterize the ethnic make-up of the school?
Melanie: I don't think it's that, it sounds, it's a shocking word to say. … Right. This is the honest truth, I think there are quite a few Pakistanis at the school, which is fine, which is great you know. I'm not a racist in the slightest, I don't really care as long as it doesn't affect me, if they're a nice person and it doesn't really matter does it what colour your skin is, […] what country they're from I don't care. What we do care about is things like the school, like everything, the whole world has gone politically correct and I think our school is quite a, like we have a racist [incident] book for example, so if a white child does anything to a Pakistani child then us as a parent and the child sign the racist book. Now in my eyes it should work both ways, but it doesn't so, I think things like that annoy you. Things like, oh we're going to stop the Christmas nativity because of that, well hang on a minute we' re in England, you know, things like oh the kids have to go off and visit the mosque, they have to take their shoes and socks off and they have to like do reading from the Quran and they have to respect it.

The school administrator confirmed that neither Christmas nor nativity plays had been stopped in the school and said ‘although we're a multi-racial school, Christmas is quite a big deal here’.^16^ What this illustrates is the power of media scares around the ‘banning of Christmas’ in the face of religious minority sensibilities, as well as the existence of mistrust around religious differences.^17^ Here we see discourse of tolerance which suggests it is at least provisional and Melanie would seem to have quite a high level of discomfort around discussing religious difference, which remains entangled with race.

In the interviews of parents from Cheadle, as well as evidence of discomfort about what was seen as a changing racial make-up of the area, there was also some discussion of white flight. Annabel had moved from Burnage, an area with a more South Asian population, to Cheadle. The reason that she gave for this move was wanting to avoid single-sex secondary schools which had been ‘a big discussion with the white parents’ (although she also acknowledged that some Asian parents did not want single-sex education). Whilst Annabel said: ‘It's not a problem for me, I mean, the way schools teach religions and how to accept other children of different nationalities.’ Nonetheless she was happier in the school in Cheadle where the majority of the children were ‘white British’ and more middle class:

I think it's good. I think because the children will be among children that are like themselves. And although it's good for them to mix with children from other classes and other ethnic backgrounds, you just gravitate towards people that are similar to you, don't you? And they're obviously going to have more chance of making good friends among children that have a similar background … It's nice for them to be among more white children.

This is clearly a different response to diversity and multiculture than the general view expressed by those interviewed in Chorlton and Whalley Range. The ‘naturalness’ of ‘gravitating towards your own’ wins out against any embrace of difference. Difference also brings with it inconvenience. Nicola explains how she felt that in Burnage her children's social networks and capital were limited. She used the example of afternoon tea invitations which were not reciprocated by her children's friends, because ‘children from big Asian families, who obviously live a different life, it's difficult, coming home for tea, it's difficult because these children cannot’ and how vegetarian food had to be provided ‘because of halal meats’.

## Conclusion

Richard Hacker has argued that multiculturalism as a concept is ‘given only a taken-for-granted commonsense meaning, impoverished both theoretically and in terms of concrete lived experience. It is a concept innocent of class’ (quoted in May 2002: 129). In this paper we have tried to argue that multiculture in terms of racialized or cultural difference is best understood within the context of a whole range of differences that people encounter in the everyday, including differences of class, religion and what some respondents in this study characterized as ‘ethical’ differences in approaches to childrearing and consumption. Despite policy-level declarations of the end of multiculturalism, in the everyday, individuals and communities, such as those created by a school, continue to negotiate cultural difference in a variety of ways. Alongside policies concerned with multiculturalism and community cohesion in schools, has also been a policy of increasing parental choice in schools. As Alexander (2007: iii) points out, there is a potential contradiction between the idea that schools can *both* be the site of multicultural citizenship and the exercise of choice. It raises questions of who should ‘shoulder the burden of integration’ and who is granted ‘the privilege of individual choice’.

This paper has attempted to shed light on parents' responses to difference as they enact school choice. What we have found is that attitudes towards and experiences of multiculture can both impact on choices and produce different ways of talking about it. So, for some parents, the perceived ethnic, religious and social make-up of school populations will impact on their choices. These choices are made in particular contexts, reflecting both the choices available and the experience of primary schools and local areas which also shapes how parents become used to talking about difference. The sense of ease with, and embrace of, multiculture varies among different groups of parents. So, for those in Whalley Range and Chorlton schools, who are already used to high levels of ethnic diversity in primary schooling, there is a sense that convivial mixing can be achieved and is desirable. However, this ease of mixing is tempered by concerns about class difference. In interviews with parents living in Cheadle, there is more of a tendency to view ethnic difference as a difference which is naturalized and which presents unbridgeable distance. At least part of this difference in responses comes from a different class habitus. Thus for the more professional middle classes in Chorlton, many of them working in the public sector, a ‘liberal’ and convivial attitude is part of their class habitus (Bourdieu, 1991). What is interesting is how this language is also shared by some ethnic minorities and migrants from a variety of class positions. For the white working- and middle-class parents living in Cheadle, there is more hesitation in both talking about and perhaps living with difference. These differences point to the need to study everyday multiculture in its context, where it is lived and to be alive to the other differences that matter.

In addition, there is a marked paucity of language with which to express these negotiations with diversity. The same terms might be used by different people to describe very different situations. Social agreement is clearly lacking on what constitutes a ‘mix’ or ‘diversity’. This poses the risk that parents, schools and policy makers may well be failing to communicate well with each other about difference and schooling. It is also important to track how cultural difference can contain ideas of both racialized or ethnic difference and religious difference which are mutually constructed and sometimes difficult to unpack. Nonetheless, this research has been able to show that, whatever the future political fate of multiculturalism, it is clear that a range of categories of difference (but particularly those of race/ethnicity, class and religion) shape how individuals and groups negotiate multiculture in considering schooling for their children.

## Notes

www.guardian.co.uk/society/2004/may/28/equality.raceintheuk (accessed 4 July 2011).

See, for example, www.prospectmagazine.co.uk/2010/09/how-multiculturalism-fails-immigrants/

www.independent.co.uk/news/uk/politics/cameron-my-war-on-muticulturalism-2205074.html (accessed 4 March 2011).

Gender clearly plays an important role in constituting a ‘mix’ in schools (where there is choice between co-educational and single-sex schools). It also can shape parents' responses to cultural difference and mix. What they desire or fear for their sons (in terms of cultural difference and mixture) may be different from what they fear or desire for their daughters. Equally, the construction of a racialized ‘other’ is also at the same time a gendered process. However, this article cannot, due to constraints of length, draw out in detail the gendered nature of these processes.

State-funded schooling in England is largely divided into ‘primary’ (usually from ages 4–10), ‘secondary’ (11–16) and sixth form (16–18).

The names of the primary schools and of the interviewees have been changed to protect anonymity.

Generally, statistics on the class background of children is not collected in schools. However, ‘free school meals’ provision, which is an income-related benefit, can serve as an indicator of deprivation (although it does not capture the deprivation of some immigrants who, depending on their residency status, are not eligible for free school meals). See Gillborn *et al*. (2012) and Gorard (2012) for further discussion.

This information comes from the relevant Ofsted reports for the schools, but is not cited in more detail to maintain anonymity. The Ofsted reports do not give detailed statistics, but overview figures as have been reported here.

With thanks to an anonymous reviewer for highlighting this issue.

One of the most likely options for parents seeking to avoid the schools of Whalley Range would be to go to Longford Primary in Chorlton. As the interview with Tej and Jas discussed below demonstrates, this ‘flight’ from particular ethnic minority concentrations is not confined only to white residents.

The majority of the interviews were with individual parents, however five interviews were conducted with both parents present.

Twelve of the women did not have paid employment and were primarily at home, several others worked part-time. One of the men interviewed on his own was also the primary carer.

In Britain, the term ‘Asian’ generally refers to those from the South-Asian sub-continent.

Weekes-Bernard (2007) also found that concern about racism was a significant consideration for many BME parents.

In Chorlton, too, some respondents expressed concern about gang culture, some of which was associated with racialized groups, but others explicitly said that the ‘gangs’ went across racialized groups. This fear of gangs also has a gendered element, in that it was more likely to be brought up by parents of boys rather than girls. It links in to the debates around ‘boys in crisis’ which are also ethnicized in particular ways (Archer, 2003).

Phone conversation.

See Gillborn (2009) for a discussion of the creation of white victimhood in the media, with specific reference to education.
